# Real-world comparative outcomes of pyrotinib-based *versus* pertuzumab-based first-line therapy in HER2-Positive advanced breast cancer: a multicenter retrospective cohort study from Xinjiang, China

**DOI:** 10.3389/fphar.2026.1943895

**Published:** 2026-10-15

**Authors:** Yimuran Hujiahemaiti, Yinping Ge, Chao Zhang, Gulisire Abdoukerim, Jing Fei, Ruifeng Qi, Yan Li, Xiaoping Ma, Zhenhui Zhao, Li Li, Dan Liu, Bingyu Li, Xiner Zhang, Bing Zhao

**Affiliations:** 1 Department of Breast Cancer, Affiliated Tumor Hospital of Xinjiang Medical University, Urumqi, Xinjiang, China; 2 Department of Oncology, Yili Kazakh Autonomous Prefecture Friendship Hospital, Yining, Xinjiang, China; 3 Department of Oncology, The First People’s Hospital of Kashgar Prefecture, Kashgar, Xinjiang, China; 4 Department of Oncology, Aksu Prefectural People’s Hospital, Aksu, Xinjiang, China; 5 Department of Oncology, The First Affiliated Hospital of Shihezi University, Shihezi, Xinjiang, China; 6 Department of Oncology, People’s Hospital of Bayingolin Mongol Autonomous Prefecture, Korla, Xinjiang, China

**Keywords:** brain metastases, diarrhea, HER2-positive breast cancer, pertuzumab, pyrotinib, real-world study

## Abstract

**Background:**

Direct comparative evidence between taxane plus trastuzumab plus pyrotinib (THPyro) and taxane plus trastuzumab plus pertuzumab (THP) as first-line therapy for HER2-positive advanced breast cancer remains limited.

**Methods:**

This multicenter retrospective real-world cohort study included 181 female patients in Xinjiang, China who initiated first-line THP or THPyro between 1 January 2020 and 30 September 2025. Propensity score matching generated 60 matched pairs. The primary endpoint was progression-free survival (PFS). Median follow-up in the matched cohort was 28.4 months.

**Results:**

After matching, baseline characteristics were well balanced. THPyro showed numerically longer median PFS than THP (20.1 vs. 17.6 months; log-rank P = 0.086), and the stratified Cox model showed a non-significant trend toward reduced progression risk (hazard ratio, 0.478; 95% confidence interval, 0.216–1.061). Restricted mean survival time (RMST) analysis demonstrated a significant 24-month PFS gain with THPyro (absolute difference, 2.41 months; P = 0.007). Objective response and disease control rates were comparable. Brain metastasis status was the most prominent potential effect modifier, with an exploratory signal observed in patients with baseline brain metastases. THPyro was associated with more diarrhea, and higher albumin level and prophylactic loperamide use were associated with a lower observed risk of grade ≥2 diarrhea.

**Conclusion:**

First-line THPyro was associated with a non-significant trend toward longer PFS and a significant 24-month RMST gain compared with THP in this retrospective matched cohort. An exploratory signal was observed among patients with baseline brain metastases, while diarrhea was more frequent and required proactive management.

## 1 Introduction

Human epidermal growth factor receptor 2 (HER2)-positive advanced breast cancer represents a biologically aggressive subtype, but its prognosis has changed substantially with the development of HER2-directed therapy. Current treatment frameworks emphasize sequential anti-HER2 strategies, with dual HER2 blockade and taxane-based chemotherapy remaining an important first-line approach in many clinical settings, while antibody-drug conjugates and small molecule tyrosine kinase inhibitors have reshaped later-line treatment landscapes (Al Sukhun et al., 2024; Cortés et al., 2022; Suppan and Balic, 2023). Nevertheless, optimal first-line treatment selection remains clinically nuanced because efficacy, toxicity, central nervous system (CNS) activity, accessibility, and regional practice patterns may differ across patient populations.

Pertuzumab plus trastuzumab and a taxane (THP) is widely recognized as a standard first-line regimen for HER2-positive metastatic breast cancer, but pyrotinib has emerged as an important alternative anti-HER2 strategy in China. Pyrotinib is an irreversible pan-HER tyrosine kinase inhibitor targeting epidermal growth factor receptor (EGFR), HER2, and HER4. In previously treated HER2-positive metastatic breast cancer, pyrotinib plus capecitabine improved progression-free survival (PFS) compared with lapatinib plus capecitabine in the phase 3 PHOEBE trial (Xu et al., 2021). More recently, the PANDORA phase 2 trial and the PHILA randomized phase 3 trial extended pyrotinib-based therapy into the first-line setting, showing promising activity when combined with trastuzumab/docetaxel or docetaxel-based chemotherapy (Ma et al., 2023; Zheng et al., 2023). Longer-term PHILA data further support the durability of clinical benefit, although gastrointestinal toxicity, particularly diarrhea, remains a key management issue (Ma et al., 2026).

Despite these advances, direct evidence comparing pyrotinib-based first-line therapy with THP in routine practice remains limited. Recent multicenter Chinese real-world studies have compared pyrotinib plus trastuzumab and a taxane (THPyro) with THP, but questions remain regarding the generalizability of these findings across regional populations, the balance between early disease control and toxicity, and the clinical subgroups most likely to benefit (Kuang et al., 2026; Liu et al., 2025). This issue is particularly relevant for patients with brain metastases, a population with substantial unmet need in HER2-positive metastatic breast cancer. Emerging real-world data suggest that pyrotinib-based therapy may have activity in patients with brain metastases, but further comparative evidence is needed (Li et al., 2025; Peng et al., 2025).

Another important consideration is toxicity management. Diarrhea is one of the most characteristic adverse events associated with HER-family tyrosine kinase inhibitors, and real-world analyses of pyrotinib-based therapy have highlighted the clinical relevance of diarrhea timing, severity, and management (Li et al., 2026). Therefore, beyond comparing efficacy and safety, clinically useful analyses should also explore treatment-effect heterogeneity and identify patients at higher risk of clinically meaningful toxicity. In this multicenter real-world study from Xinjiang, China, we compared first-line THPyro with THP in patients with HER2-positive advanced breast cancer using propensity score-based methods, and further explored subgroup-specific treatment effects and exploratory models for patients with brain metastases and THPyro-associated diarrhea.

## 2 Materials and methods

### 2.1 Study design

This retrospective, multicenter, real-world cohort study was conducted at six hospitals in Xinjiang, China, led by the Affiliated Tumor Hospital of Xinjiang Medical University. The participating hospitals were the Affiliated Tumor Hospital of Xinjiang Medical University, Yili Kazakh Autonomous Prefecture Friendship Hospital, the First People’s Hospital of Kashgar, Aksu Regional People’s Hospital, the First Affiliated Hospital of Shihezi University, and Bayingolin Mongol Autonomous Prefecture People’s Hospital. Among the 181 patients included in the final analytic cohort, 152 were enrolled from the Affiliated Tumor Hospital of Xinjiang Medical University, including 74 patients in the THPyro group and 78 patients in the THP group; the remaining five hospitals contributed 29 patients, including 17 in the THPyro group and 12 in the THP group.

The study was conducted in accordance with the Declaration of Helsinki and applicable local ethical and regulatory requirements. The study protocol was approved by the Ethics Review Committee of Xinjiang Medical University Cancer Hospital (approval No. K-2026098), and the requirement for written informed consent was waived because of the retrospective design. Clinical trial registration was not required for this retrospective observational study.

The study was aligned with a target-trial framework to improve the transparency of the observational comparison. The treatment strategies of interest were first-line THP, defined as a taxane plus trastuzumab plus pertuzumab, and first-line THPyro, defined as a taxane plus trastuzumab plus pyrotinib. Time zero was defined as the date of first administration of THP or THPyro as the standard first-line regimen for recurrent, metastatic, or initially unresectable advanced disease. The data extraction cutoff date and outcome-event follow-up cutoff date were both 31 January 2026.

### 2.2 Participants

Female patients with HER2-positive advanced breast cancer who initiated first-line THP or THPyro between 1 January 2020 and 30 September 2025 were identified through systematic screening of electronic medical records at each participating center. Consecutive patients meeting the prespecified eligibility criteria during the study period were included. Patients were eligible if they were 18 years or older; had histologically confirmed invasive breast cancer with postoperative recurrence, metastatic disease, or initially diagnosed unresectable locally advanced or metastatic disease; had HER2-positive disease, defined as immunohistochemistry 3+ or immunohistochemistry 2+ with fluorescence *in situ* hybridization-confirmed erythroblastic oncogene B2 amplification (HER2/chromosome enumerate probe 17 ratio≥2.0); received first-line THP or THPyro after recurrence, metastasis, or initial diagnosis of advanced disease; had at least one measurable lesion according to Response Evaluation Criteria in Solid Tumors (RECIST) version 1.1; had an expected survival of at least 3 months; had organ function considered adequate for treatment according to routine clinical assessment; and had complete and traceable clinical data during treatment.

First-line therapy was defined as the first THP or THPyro regimen administered during the recurrent, metastatic, or initially advanced disease stage. Prior neoadjuvant or adjuvant trastuzumab was permitted if THP or THPyro was the first anti-HER2 regimen used after recurrence or metastasis. The taxane component included liposomal paclitaxel, docetaxel, albumin-bound paclitaxel, or paclitaxel injection. Brain metastasis was defined as CNS metastasis confirmed by brain magnetic resonance imaging or contrast-enhanced computed tomography. Patients with stable or asymptomatic brain metastases were eligible, whereas patients with active or clinically symptomatic brain metastases were excluded. Baseline measurable disease was required to be documented by imaging performed within 4 weeks before initiation of study treatment.

The exclusion criteria were male breast cancer; baseline left ventricular ejection fraction below 55%; other primary malignancies; severe non-cancer-related comorbidities that substantially increased treatment risk or interfered with survival assessment; pregnancy or lactation; prior exposure to pertuzumab or HER2-targeted tyrosine kinase inhibitors, such as pyrotinib or lapatinib, for recurrent or metastatic disease before initiation of first-line advanced-stage therapy; Eastern Cooperative Oncology Group (ECOG) performance status greater than 3; loss to follow-up before the first efficacy assessment; or treatment with other anti-HER2 agents, including trastuzumab monotherapy or antibody-drug conjugates, as the study regimen. Severe non-cancer-related comorbidities included New York Heart Association class III-IV cardiac dysfunction, unstable angina, myocardial infarction, or stroke within 6 months before treatment initiation, severe arrhythmia requiring medical control, severe respiratory, neurologic, or other major organ-system disease, active severe infection, or uncontrolled psychiatric illness that could interfere with treatment adherence or follow-up.

Patients with incomplete key clinical data were excluded to ensure the integrity of the analytic cohort. Incomplete data were defined as absence of baseline imaging records required for disease staging or response assessment, missing treatment start or end dates precluding definition of time zero or treatment duration, or absence of efficacy assessment or survival follow-up records preventing endpoint determination. These patients were not included in the primary analytic cohort to reduce measurement and information bias related to incomplete retrospective records.

### 2.3 Treatment and follow-up

Patients received first-line THP or THPyro after recurrence, metastasis, or initial diagnosis of advanced disease. Because this was a retrospective real-world study, regimen selection, drug administration, dose modification, supportive care, and follow-up schedules were determined by the treating physicians at each center according to routine clinical practice. The study did not interfere with clinical decision-making. Treatment-related information extracted from medical records included treatment start date, regimen components, drug dose, administration cycle, treatment line, treatment duration, treatment stop date, and reason for treatment discontinuation. Reasons for treatment discontinuation included disease progression, adverse events, patient preference, financial reasons, and loss to follow-up when documented.

Supportive care was determined by the treating physician according to patient condition and local practice. Supportive measures extracted from records included antidiarrheal treatment, antiemetic treatment, leukocyte-supportive treatment, transfusion or erythropoiesis-stimulating treatment, hepatoprotective treatment, anti-infective treatment, analgesic treatment, bone-protective treatment, and other symptomatic management. Antidiarrheal treatment, particularly prophylactic loperamide use, was specifically collected for safety and diarrhea risk analyses.

Tumor response was assessed according to RECIST version 1.1. Baseline imaging was defined as systemic or lesion-specific imaging completed within 28 days before treatment initiation. The first post-treatment efficacy assessment was generally performed 6–8 weeks after treatment initiation, with an allowed window of ± 2 weeks. Subsequent assessments followed each center’s routine clinical practice. If clinical symptoms suggested disease progression and prompted earlier imaging, that imaging result could be used for response or progression assessment. The data extraction cutoff date and outcome-event follow-up cutoff date were both 31 January 2026.

### 2.4 Data collection and safety assessment

Clinical data were retrospectively extracted by designated investigators at each participating center from electronic medical records, imaging reports, pathology reports, laboratory systems, and medication records using a standardized data collection form. Extracted variables included age, menopausal status, ECOG performance status, hormone-receptor status, Ki-67 expression, initial tumor-node-metastasis (TNM) stage, prior neoadjuvant or adjuvant trastuzumab therapy, metastatic burden, metastatic sites, baseline laboratory indicators, treatment regimen, dose modification, adverse events, response assessments, disease progression date, and survival status. Baseline laboratory indicators were defined as the most recent available results before treatment initiation, preferably within 14 days before treatment and, when unavailable, within 28 days before treatment if no major clinical change had occurred. These indicators included blood counts, liver function, renal function, and treatment-safety-related markers, such as white blood cell count, neutrophil count, hemoglobin, platelet count, alanine aminotransferase, aspartate aminotransferase, total bilirubin, and albumin.

Before data collection, the study team established a unified data dictionary and variable definitions. Investigators involved in data entry were trained according to the standardized definitions. After data entry, the coordinating center performed data consistency checks, and any abnormal values, inconsistent information, or missing key variables were resolved by reviewing original medical records, imaging reports, and laboratory records. Key endpoints, including response assessment, disease progression date, and adverse event grade, were reviewed independently by at least two investigators. Disagreements were resolved by discussion and, when necessary, adjudication by senior investigators.

This study did not establish an independent central imaging review committee. Tumor response assessments were based on imaging reports generated during routine care at each center and were retrospectively reviewed by study investigators according to RECIST version 1.1. For cases with incomplete imaging descriptions, ambiguous staging information, or inconsistency with the clinical course, the study team reviewed the available imaging reports, relevant laboratory results, and follow-up records to determine the final response or progression status. The disease progression date was defined as the date of the first imaging examination or clinical record documenting definite disease progression. For imaging progression, the date of the imaging examination showing progression, rather than the report issue date, was used. When no clear imaging progression was available but medical records documented treatment change, treatment termination, or palliative care because of clinical deterioration, the clinical progression date was used.

Brain metastasis diagnosis and follow-up were based primarily on contrast-enhanced brain magnetic resonance imaging. Contrast-enhanced brain computed tomography was accepted when magnetic resonance imaging was unavailable or when computed tomography was used in routine clinical practice. Brain imaging was not mandatory for all patients and was performed according to baseline brain metastasis status, neurologic symptoms, prior CNS history, and local clinical practice. For patients with baseline brain metastases, brain imaging follow-up was performed according to the treating physician’s judgment. Additional CNS-related and disease-burden variables were extracted when available to support descriptive evaluation of the brain-metastasis subgroup, including age category, ECOG performance status, hormone-receptor status, Ki-67 expression, number of metastatic sites, receipt of brain-directed radiotherapy, and number of intracranial lesions. The number of intracranial lesions was categorized as 1–2 lesions or ≥3 lesions according to baseline brain imaging records. Data on intracranial lesion size were not consistently available in the retrospective records and therefore were not included. Surgical local treatment for advanced-stage disease was rare in this cohort and was not summarized as a baseline variable.

RECIST version 1.1 was used as the main framework for response evaluation. Patients with at least one measurable lesion were included in the primary objective response rate (ORR) and disease control rate (DCR) analyses. Patients with only non-measurable lesions were evaluated according to non-target lesion principles, imaging reports, and clinical judgment but were not included in the primary objective response analysis. For brain metastases, lesions meeting RECIST version 1.1 measurability criteria could be used for CNS response evaluation; otherwise, they were assessed as non-target lesions. Computed tomography or magnetic resonance imaging was prioritized for lung, brain, deep visceral lesions, or target lesions requiring precise measurement. Ultrasonography was used only as an auxiliary modality for selected superficial or abdominal lesions. If different imaging modalities produced inconsistent findings, computed tomography or magnetic resonance imaging and the overall clinical assessment were prioritized.

Adverse events were evaluated from clinical documentation and laboratory findings during treatment and were graded according to the National Cancer Institute Common Terminology Criteria for Adverse Events (CTCAE) version 5.0. The adverse event observation window extended from the first study treatment administration to 30 days after the last study treatment dose or to the start of a new antitumor treatment, whichever occurred first. Diarrhea was defined as a treatment-related diarrhea event occurring during study treatment or within the adverse event observation window. THPyro-associated grade 2 or higher diarrhea was defined as diarrhea judged by the investigator to be possibly, probably, or definitely related to THPyro and graded as two or higher according to CTCAE version 5.0. Grade 3 or higher diarrhea was defined using the same causality criteria and was analyzed as an exploratory safety endpoint.

### 2.5 Study endpoints

The primary endpoint was PFS, defined as the interval from time zero to the first documented disease progression or death from any cause, whichever occurred first. Patients without documented progression or death by 31 January 2026 were censored on 31 January 2026. Patients lost to follow-up before 31 January 2026 without documented progression were censored at the last date confirmed to be progression-free. Patients lost to follow-up without documented progression were censored at the last date confirmed to be progression-free. Secondary endpoints included ORR, DCR, overall survival (OS), and adverse events. OS was defined as the interval from time zero to death from any cause. Patients alive on 31 January 2026 were censored on 31 January 2026, and patients lost to follow-up before 31 January 2026 were censored at the last date they were confirmed to be alive.

ORR was defined as the proportion of patients achieving complete response or partial response, and DCR was defined as the proportion achieving complete response, partial response, or stable disease. The ORR and DCR evaluation set included patients who received at least one study treatment, had measurable baseline disease, and completed at least one post-treatment response assessment. Patients without post-treatment imaging assessment who discontinued treatment because of definite disease progression or death were classified as non-responders or as having progressive disease when supported by available clinical and imaging records. Dose modification and treatment discontinuation data included dose reduction, dose delay, temporary interruption, and permanent discontinuation, together with documented reasons such as adverse events, disease progression, patient preference, comorbidities, financial reasons, and loss to follow-up.

### 2.6 Statistical analysis

All statistical analyses were performed using R software (version 4.5.2). Continuous variables were summarized as mean ± standard deviation or median with interquartile range, and categorical variables were summarized as counts and percentages. Between-group comparisons were performed using t tests or Mann-Whitney U tests for continuous variables and chi-square or Fisher exact tests for categorical variables, as appropriate. All tests were two-sided, and P < 0.05 was considered statistically significant unless otherwise specified. Missing data were summarized descriptively. Missing baseline and safety variables were not imputed and were analyzed using available data. For efficacy and survival variables, missing dates or event information were verified through traceable clinical records, imaging reports, and follow-up records. If key outcomes could not be confirmed, the patient was censored or excluded from the specific endpoint analysis, as appropriate.

To reduce baseline imbalance, 1:1 nearest-neighbor propensity score matching (PSM) without replacement was performed using a caliper width of 0.2 of the standard deviation of the logit of the propensity score. Propensity scores were estimated using a multivariable logistic regression model incorporating baseline clinical and pathological variables available before treatment selection, including age, menopausal status, ECOG performance status, hormone receptor status, Ki-67 expression, initial TNM stage, prior neoadjuvant or adjuvant trastuzumab therapy, visceral metastasis, brain metastasis, bone metastasis, other distant metastatic sites, metastatic burden, and baseline laboratory indicators. Covariate balance was assessed using standardized mean differences and Love plots, with standardized mean differences below 0.1 indicating adequate balance.

Tumor response outcomes were compared using chi-square or Fisher exact tests before matching and conditional logistic regression after matching. PFS and OS were estimated using the Kaplan-Meier method and compared using the log-rank test. In the matched cohort, treatment effects on PFS were evaluated using stratified Cox proportional hazards models with matched subclasses as strata. The proportional hazards assumption was assessed using Schoenfeld residuals. To assess the statistical power of the matched cohort to detect the observed treatment effect, a *post hoc* power analysis was performed based on the Cox proportional-hazards framework. Using the observed HR of 0.478 from the conditional Cox model, with a two-sided α of 0.05 and equal allocation between the two treatment groups, 58 and 78 PFS events were required to achieve 80% and 90% power, respectively. In the matched cohort, 87 PFS events were observed among 120 patients (60 per group), corresponding to an estimated nominal power of 93.1%. When potential non-proportionality was identified, time-dependent Cox models were used to assess treatment-by-time interaction, and restricted mean survival time (RMST) analysis was used as a complementary method not requiring the proportional hazards assumption. RMST analysis used a prespecified truncation time of 24 months, selected according to the follow-up distribution, event accumulation within 24 months, stability of survival curve estimation, and clinical interpretability for early-to-intermediate disease control. Standard errors, 95% confidence intervals (CI), and P values for RMST differences were estimated using 1,000 bootstrap resamples.

Treatment-effect heterogeneity was explored using inverse probability of treatment weighting (IPTW) in the full cohort. Stabilized weights were used. For treated patients, the stabilized weight was calculated as the marginal probability of receiving the study treatment divided by the individual propensity score; for control patients, the stabilized weight was calculated as the marginal probability of not receiving the study treatment divided by one minus the individual propensity score. To reduce the influence of extreme weights, weights were truncated at the 5th and 95th percentiles in the main IPTW analysis. Robust standard errors were used for weighted treatment-effect estimates, and treatment-by-subgroup interaction P values were obtained from robust Wald tests.

Because this was a multicenter real-world study, data from participating centers were combined after standardized data extraction and quality control using unified variable definitions and response assessment principles. Study center was not included as a covariate, stratification factor, or clustering variable in the main models, and center-specific effects were not separately evaluated. Safety analyses involving multiple adverse event comparisons were adjusted using the false discovery rate (FDR) approach. Other secondary endpoints, subgroup analyses, and prediction model analyses were considered exploratory and hypothesis-generating, and their P values were interpreted descriptively rather than as confirmatory evidence. Accordingly, center-level confounding could not be fully addressed in the current analysis.

Exploratory models were developed for PFS among patients with brain metastases and for THPyro-associated grade 2 or higher diarrhea. Candidate variables were limited according to event counts, clinical relevance, and variable completeness to reduce overfitting. For the brain metastasis PFS model, events were defined as disease progression or death. Least absolute shrinkage and selection operator (LASSO) Cox regression was used for variable selection, and selected variables were entered into a multivariable Cox model to construct a nomogram for 12- and 18-month PFS. For THPyro-associated diarrhea, events were defined as grade 2 or higher THPyro-associated diarrhea. A multivariable logistic regression model was developed, and grade 3 or higher diarrhea was explored using Firth penalized logistic regression because severe events were infrequent. Model discrimination, calibration, decision curve analysis, internal validation, and optimism correction using 1,000 bootstrap resamples are described in the Supplementary Methods. No independent external validation cohort was available.

## 3 Results

### 3.1 Patient selection and baseline characteristics

Between 1 January 2020 and 30 September 2025, 214 patients with HER2-positive advanced breast cancer who initiated first-line THP or THPyro were screened, and 33 were excluded, including 19 patients who did not receive a standard study regimen, six who discontinued treatment for financial reasons, five who were lost to follow-up before the first efficacy assessment, and 3 with incomplete clinical records. Among the excluded patients, seven were from the Affiliated Tumor Hospital of Xinjiang Medical University and 26 were from the other five participating hospitals. Finally, 181 patients were included in the full analysis set, including 91 treated with THPyro and 90 treated with THP. After 1:1 PSM, 120 patients were retained, with 60 patients in each treatment group (Figure 1). The median follow-up in the matched cohort was 28.4 months.

**FIGURE 1:**
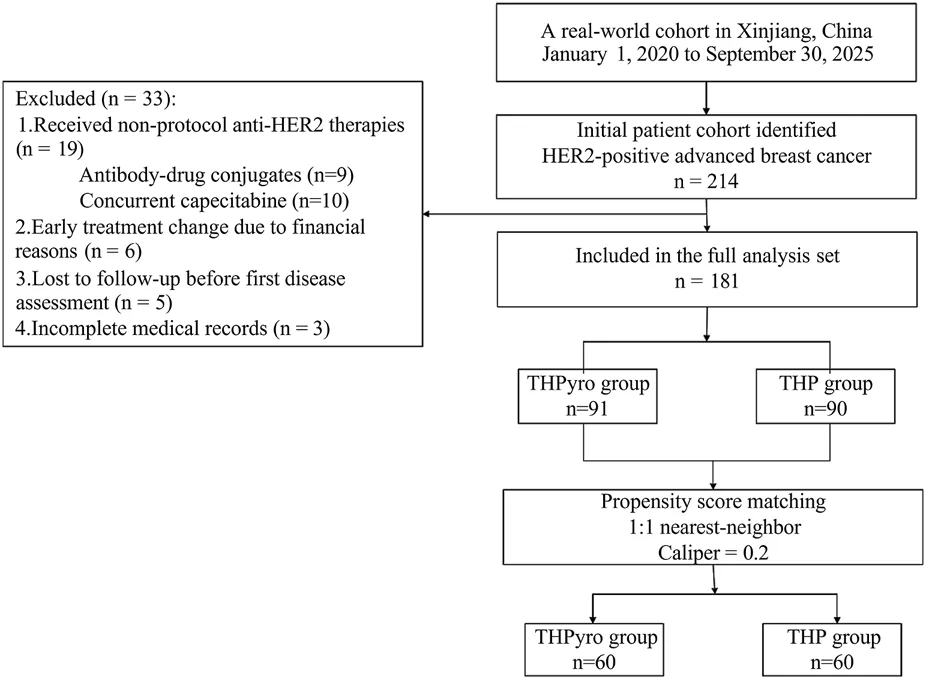
Patient selection and PSM flow diagram.

Before matching, clinically relevant baseline differences were observed, including age, Ki-67 status, visceral metastasis, and brain metastasis. After matching, these imbalances were largely attenuated, and the two groups were well balanced across prespecified clinicopathological variables (Table 1). Standardized mean differences after matching further supported adequate covariate balance (Supplementary Figure S1).

**TABLE 1:** Baseline patient characteristics before and after PSM.

Characteristics	Before matching				After matching			
	Total (N = 181)	THPyro (N = 91)	THP (N = 90)	*P* value	Total (N = 120)	THPyro (N = 60)	THP (N = 60)	P value
Age, years	51.6 ± 9.6	53.1 ± 9.0	50.1 ± 9.9	0.038	51.5 ± 9.5	51.3 ± 9.1	51.8 ± 10.0	0.760
Age	​	​	​	0.057	​	​	​	1.000
< 55	107 (59.1)	47 (51.6)	60 (66.7)	​	72 (60.0)	36 (60.0)	36 (60.0)	​
≥ 55	74 (40.9)	44 (48.4)	30 (33.3)	​	48 (40.0)	24 (40.0)	24 (40.0)	​
Menopause status	​	​	​	0.264	​	​	​	0.713
Premenopausal	76 (42.0)	34 (37.4)	42 (46.7)	​	53 (44.2)	28 (46.7)	25 (41.7)	​
Postmenopausal	105 (58.0)	57 (62.6)	48 (53.3)	​	67 (55.8)	32 (53.3)	35 (58.3)	​
ECOG performance status score	​	​	​	0.846	​	​	​	0.804
0	88 (48.6)	44 (48.4)	44 (48.9)	​	59 (49.2)	30 (50.0)	29 (48.3)	​
1	60 (33.1)	29 (31.9)	31 (34.4)	​	41 (34.2)	19 (31.7)	22 (36.7)	​
≥ 2	33 (18.2)	18 (19.8)	15 (16.7)	​	20 (16.7)	11 (18.3)	9 (15.0)	​
Hormone-receptor status	​	​	​	0.712	​	​	​	0.584
Positive	87 (48.1)	42 (46.2)	45 (50.0)	​	62 (51.7)	29 (48.3)	33 (55.0)	​
Negative	94 (51.9)	49 (53.8)	45 (50.0)	​	58 (48.3)	31 (51.7)	27 (45.0)	​
Ki-67, n (%)	​	​	​	0.024	​	​	​	1.000
≤ 20%	46 (25.4)	16 (17.6)	30 (33.3)	​	29 (24.2)	15 (25.0)	14 (23.3)	​
> 20%	135 (74.6)	75 (82.4)	60 (66.7)	​	91 (75.8)	45 (75.0)	46 (76.7)	​
Disease characteristic	​	​	​	0.670	​	​	​	1.000
De novo stage IV	123 (68.0)	60 (65.9)	63 (70.0)	​	82 (68.3)	41 (68.3)	41 (68.3)	​
Recurrent disease	58 (32.0)	31 (34.1)	27 (30.0)	​	38 (31.7)	19 (31.7)	19 (31.7)	​
TNM-Stage at initial diagnosis	​	​	​	0.576	​	​	​	0.990
I	4 (2.2)	2 (2.2)	2 (2.2)	​	2 (1.7)	1 (1.7)	1 (1.7)	​
II	21 (11.6)	9 (9.9)	12 (13.3)	​	15 (12.5)	8 (13.3)	7 (11.7)	​
III	33 (18.2)	20 (22.0)	13 (14.4)	​	21 (17.5)	10 (16.7)	11 (18.3)	​
IV	123 (68.0)	60 (65.9)	63 (70.0)	​	82 (68.3)	41 (68.3)	41 (68.3)	​
Previous trastuzumab therapy in adjuvant/neoadjuvant phase	​	​	​	0.776	​	​	​	1.000
No	130 (71.8)	64 (70.3)	66 (73.3)	​	87 (72.5)	44 (73.3)	43 (71.7)	​
Yes	51 (28.2)	27 (29.7)	24 (26.7)	​	33 (27.5)	16 (26.7)	17 (28.3)	​
Metastatic sites								
Visceral	133 (73.5)	60 (65.9)	73 (81.1)	0.032	89 (74.2)	42 (70.0)	47 (78.3)	0.404
Brain	58 (32.0)	38 (41.8)	20 (22.2)	0.008	38 (31.7)	20 (33.3)	18 (30.0)	0.844
Bone	97 (53.6)	48 (52.7)	49 (54.4)	0.936	66 (55.0)	31 (51.7)	35 (58.3)	0.582
Other	80 (44.2)	36 (39.6)	44 (48.9)	0.265	56 (46.7)	28 (46.7)	28 (46.7)	1.000

THPyro, taxanes plus trastuzumab plus pyrotinib; THP, taxanes plus trastuzumab plus pertuzumab; ECOG, Eastern Cooperative Oncology Group; TNM, tumor–node–metastasis; PSM, propensity score matching.

Data are expressed as numbers (percentages) for categorical variables and mean ± standard deviation (SD) for continuous variables. Percentages may not sum to 100% due to rounding. Metastatic sites were not mutually exclusive, patients could present with more than one metastatic site.

For statistical comparisons, P values were computed using the chi-square test or Fisher's exact test for categorical variables and the Student’s t test for continuous variables, as appropriate. All tests were two-sided, with statistical significance defined as P < 0.05. All values, except P values, were rounded to one decimal place.

### 3.2 PFS and tumor response

THPyro showed a numerical trend toward longer PFS than THP both before and after matching (Figures 2A,B). In the matched cohort, median PFS was 20.1 months (95% CI, 19.1–22.2) with THPyro and 17.6 months (95% CI, 14.8–20.8) with THP (log-rank P = 0.086; Table 2). The largest separation was observed at 12 months, when PFS rates were 96.7% and 81.6%, respectively, corresponding to an absolute difference of 15.1 percentage points; by 24 months, PFS rates were similar between groups (25.0% vs. 25.4%; Supplementary Table S1).

**FIGURE 2:**
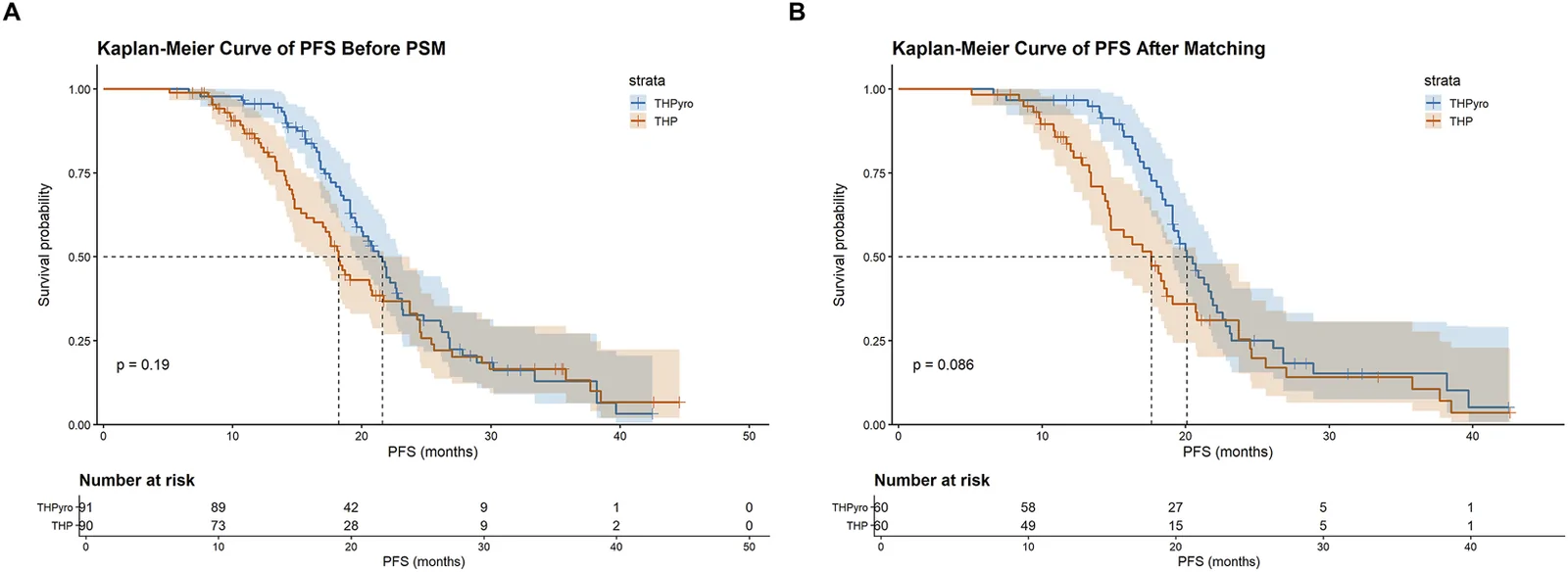
Kaplan–Meier curves for PFS before and after PSM. **(A)** PFS before PSM in the full cohort. **(B)** PFS after PSM in the matched cohort. Shaded areas indicate 95% CIs. Tick marks indicate censored observations. P values were calculated using the log-rank test. PFS, progression-free survival; PSM, propensity score matching; THPyro, taxane plus trastuzumab plus pyrotinib; THP, taxane plus trastuzumab plus pertuzumab.

**TABLE 2:** Evaluation of efficacy.

Variable	Before PSM				After PSM			
	THPyro (N = 91)	THP (N = 90)	P value	OR (95% CI)	THPyro (N = 60)	THP (N = 60)	P value	OR (95% CI)
Median PFS (months)	21.60 (19.60–23.10)	18.2 (17.00–23.70)	0.187	​	20.10 (19.10–22.20)	17.60 (14.80–20.80)	0.086	​
Events	63 (69.2)	60 (66.7)	​	​	45 (75.0)	42 (70.0)	​	​
Best overall response								
Complete response	4 (4.4)	4 (4.4)	​	​	2 (3.3)	4 (6.7)	​	​
Partial response	38 (41.8)	38 (42.2)	​	​	23 (38.3)	23 (38.3)	​	​
Stable disease	48 (52.7)	45 (50.0)	​	​	34 (56.7)	31 (51.7)	​	​
Progressive disease	1 (1.1)	3 (3.3)	​	​	1 (1.7)	2 (3.3)	​	​
ORR	42 (46.2)	42 (46.7)	0.945	0.980 (0.545–1.759)	25 (41.7)	27 (45.0)	0.706	0.867 (0.412–1.821)
DCR	90 (98.9)	87 (96.7)	0.368	3.103 (0.389–63.397)	59 (98.3)	58 (96.7)	0.571	2.000 (0.181-22.057)

PFS, progression-free survival; ORR, objective response rate; DCR, disease control rate; PSM, propensity score matching; THPyro, taxanes plus trastuzumab plus pyrotinib; THP, taxanes plus trastuzumab plus pertuzumab; OR, odds ratio; CI, confidence interval; ORR, objective response rate; DCR, disease control rate.

Values are presented as median (95% CI) or n (%).

P values for PFS were calculated using the log-rank test.

P values for ORR/DCR were calculated using chi-square or Fisher’s exact test before PSM and conditional logistic regression after PSM.

Tumor response outcomes were comparable between groups. After matching, ORR was 41.7% with THPyro and 45.0% with THP [odds ratio (OR), 0.867; 95% CI, 0.412–1.821; P = 0.706], and DCR was 98.3% and 96.7%, respectively (P = 0.571; Table 2). These findings suggest that the observed advantage of THPyro may be reflected more in prolonging PFS-related disease control than in increasing the probability of initial tumor shrinkage.

### 3.3 Time-dependent treatment effect, RMST analysis, and OS

In the matched cohort, the stratified Cox model showed a non-significant trend toward a lower risk of progression with THPyro [hazard ratio (HR), 0.478; 95% CI, 0.216–1.061; P = 0.070]. Because the proportional hazards assumption was mildly violated (global Schoenfeld test P = 0.045), a time-varying Cox model and RMST analysis were performed. The time-varying model did not show a statistically significant treatment-by-time interaction (P = 0.299), but the estimated curve was consistent with attenuation of early benefit over time (Supplementary Table S2; Supplementary Figure S2).

At the prespecified 24-month time horizon, RMST was 19.85 months with THPyro and 17.43 months with THP, corresponding to an absolute gain of 2.41 months (95% CI, 0.71–4.13; P = 0.007). Conventional time-to-event analyses therefore showed a non-significant trend favoring THPyro, whereas the prespecified 24-month RMST analysis suggested an early absolute PFS gain (Supplementary Figures S3–S5).

OS data remained immature, with only 20 deaths before matching and 13 deaths after matching. OS was therefore analyzed as an exploratory and underpowered endpoint. No significant OS difference was observed before matching (log-rank P = 0.108) or after matching (log-rank P = 0.376). Detailed OS rates are shown in Supplementary Table S3.

### 3.4 Sensitivity and subgroup analyses

In the IPTW sensitivity analysis, covariate balance improved after weighting (Supplementary Figure S6), and the direction of treatment effect was consistent with the matched analysis.

Exploratory subgroup analysis identified brain metastasis status as the most prominent potential effect modifier (Figure 3). THPyro was associated with a lower risk of progression among patients with baseline brain metastases (HR, 0.156; 95% CI, 0.074–0.327), but not among those without brain metastases (HR, 0.943; 95% CI, 0.595–1.494; P for interaction <0.001). Other subgroup signals favored THPyro in selected clinical strata, but interaction tests were not significant; therefore, these findings should be considered hypothesis-generating (Supplementary Table S4).

**FIGURE 3:**
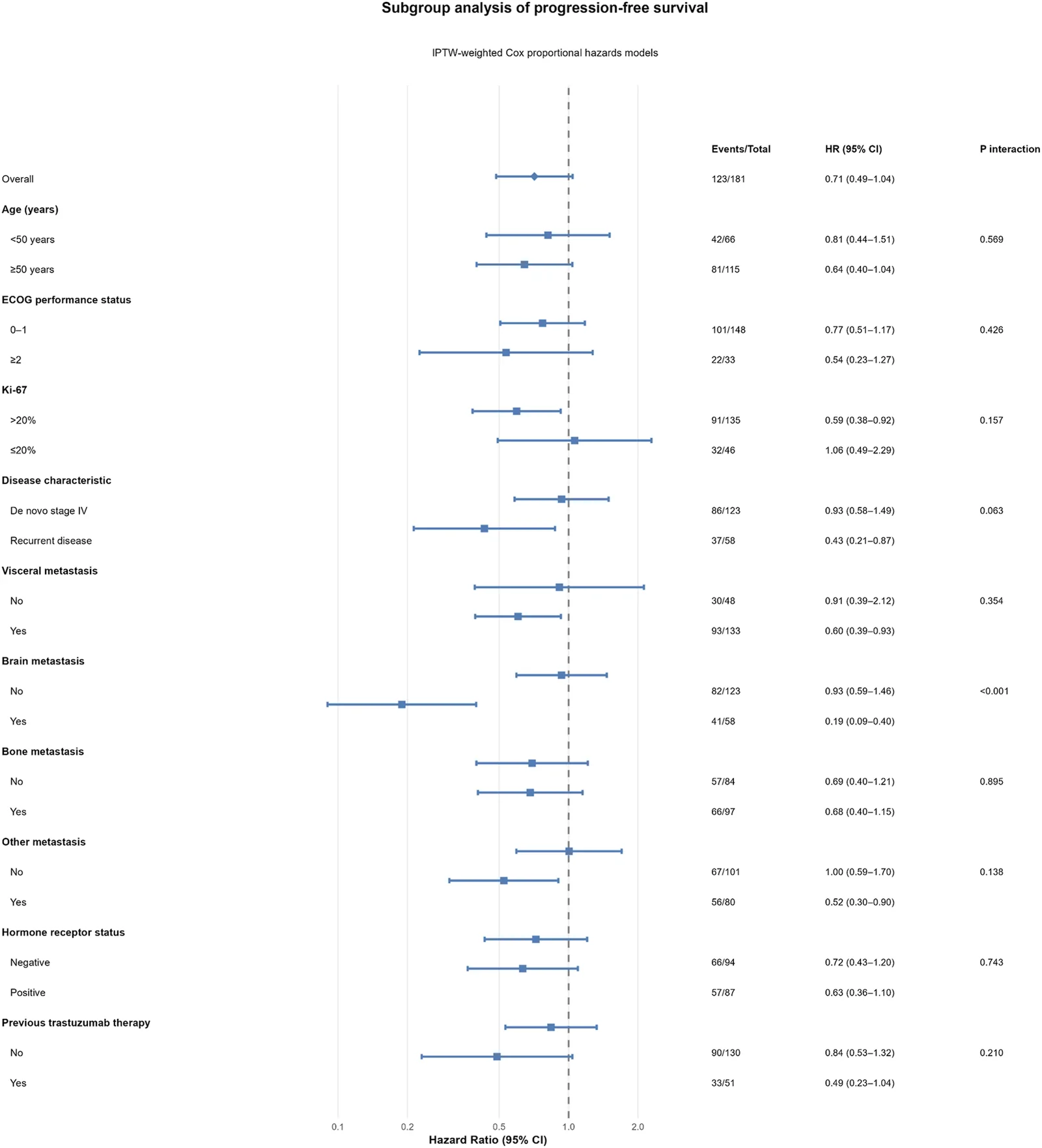
Subgroup analysis of PFS in the IPTW-weighted cohort.

To further contextualize the exploratory signal observed in patients with baseline brain metastases, baseline characteristics of this subgroup were summarized by treatment group (Supplementary Table S5). Among 58 patients with baseline brain metastases, 38 received THPyro and 20 received THP. Age category, ECOG performance status, hormone-receptor status, Ki-67 expression, and PFS event frequency were broadly comparable between groups. However, potentially relevant imbalances were observed. Brain-directed radiotherapy was more frequent in the THPyro group, whereas ≥3 intracranial lesions and ≥3 metastatic sites were numerically more frequent in the THP group.

To assess the robustness of the observed treatment effect after accounting for potential baseline imbalances in brain-directed radiotherapy, number of intracranial lesions, and number of metastatic sites, an additional sensitivity analysis was performed. After adjustment, the estimated treatment effect among patients with baseline brain metastases remained similar (adjusted HR, 0.163; 95% CI, 0.085–0.312), compared with an HR of 0.156 (95% CI, 0.074–0.327) in the original IPTW analysis. The treatment-by-brain-metastasis interaction also remained statistically significant (adjusted P for interaction <0.001), supporting the robustness of the exploratory interaction signal (Supplementary Table S6). As an additional supportive analysis, we restricted the analysis to the 58 patients with baseline brain metastases and adjusted for brain-directed radiotherapy, number of intracranial lesions, and number of metastatic sites in a multivariable Cox model. The treatment effect remained directionally consistent (adjusted HR, 0.092; 95% CI, 0.036–0.233; P < 0.001). An IPTW-weighted analysis restricted to this subgroup yielded a similar estimate (HR, 0.099; 95% CI, 0.036–0.273; P < 0.001) (Supplementary Table S7).

### 3.5 Treatment-related adverse events

The safety profile differed between regimens, with diarrhea being the most distinctive adverse event associated with THPyro (Table 3). After matching, any-grade diarrhea occurred in 66.7% of patients receiving THPyro and 20.0% receiving THP (OR, 7.84; 95% CI, 3.25–20.10; P < 0.001; FDR-adjusted P < 0.001). Grade≥3 diarrhea occurred in 10.0% of patients in the THPyro group and in none of the patients in the THP group (P = 0.027; FDR-adjusted P = 0.356).

**TABLE 3:** Comparison of adverse events (all grades and grade ≥3) between THPyro and THP groups before and after PSM.

Adverse events	PSM status	All grades					Grade ≥3				
		THPyro (N=91/60)	THP (N=90/60)	OR (95% CI)	P value	FDR P	THPyro (N=91/60)	THP (N=90/60)	OR (95% CI)	P value	FDR P
Leukopenia	Before	40 (44.0)	48 (53.3)	0.69 (0.37–1.28)	0.235	0.383	6 (6.6)	10 (11.1)	0.57 (0.16–1.81)	0.308	0.501
	After	27 (45.0)	30 (50.0)	0.82 (0.38–1.78)	0.715	0.845	4 (6.7)	7 (11.7)	0.54 (0.11–2.28)	0.529	0.625
Neutropenia	Before	24 (26.4)	45 (50.0)	0.36 (0.18–0.70)	0.001	0.008	5 (5.5)	11 (12.2)	0.42 (0.11–1.38)	0.124	0.324
	After	16 (26.7)	27 (45.0)	0.45 (0.19–1.02)	0.056	0.244	4 (6.7)	8 (13.3)	0.47 (0.10–1.87)	0.362	0.625
Anemia	Before	30 (33.0)	44 (48.9)	0.52 (0.27–0.98)	0.035	0.138	5 (5.5)	8 (8.9)	0.60 (0.15–2.17)	0.405	0.526
	After	24 (40.0)	31 (51.7)	0.63 (0.28–1.37)	0.272	0.550	3 (5.0)	6 (10.0)	0.48 (0.07–2.37)	0.491	0.625
Thrombocytopenia	Before	15 (16.5)	26 (28.9)	0.49 (0.22–1.05)	0.052	0.138	1 (1.1)	5 (5.6)	0.19 (0.00–1.75)	0.118	0.324
	After	12 (20.0)	20 (33.3)	0.50 (0.20–1.23)	0.148	0.384	1 (1.7)	4 (6.7)	0.24 (0.00–2.52)	0.364	0.625
AST increased	Before	36 (39.6)	38 (42.2)	0.90 (0.47–1.69)	0.763	0.827	5 (5.5)	9 (10.0)	0.53 (0.13–1.83)	0.281	0.501
	After	24 (40.0)	25 (41.7)	0.93 (0.42–2.06)	1.000	1.000	3 (5.0)	7 (11.7)	0.40 (0.06–1.87)	0.322	0.625
ALT increased	Before	30 (33.0)	34 (37.8)	0.81 (0.42–1.56)	0.536	0.697	3 (3.3)	10 (11.1)	0.27 (0.05–1.12)	0.048	0.313
	After	20 (33.3)	22 (36.7)	0.86 (0.38–1.96)	0.848	0.919	2 (3.3)	7 (11.7)	0.26 (0.03–1.47)	0.163	0.625
Diarrhea	Before	63 (69.2)	19 (21.1)	8.29 (4.08–17.51)	<0.001	<0.001	14 (15.4)	1 (1.1)	16.00 (2.33–689.47)	0.001	0.008
	After	40 (66.7)	12 (20.0)	7.84 (3.25–20.10)	<0.001	<0.001	6 (10.0)	0 (0.0)	​	0.027	0.356
Nausea/Anorexia	Before	59 (64.8)	49 (54.4)	1.54 (0.81–2.93)	0.174	0.324	4 (4.4)	2 (2.2)	2.02 (0.28–22.82)	0.682	0.806
	After	41 (68.3)	31 (51.7)	2.01 (0.90–4.55)	0.093	0.302	1 (1.7)	1 (1.7)	1.00 (0.01–79.76)	1.000	1.000
Vomiting	Before	34 (37.4)	28 (31.1)	1.32 (0.68–2.57)	0.434	0.627	5 (5.5)	5 (5.6)	0.99 (0.22–4.46)	1.000	1.000
	After	24 (40.0)	18 (30.0)	1.55 (0.68–3.56)	0.339	0.550	0 (0.0)	3 (5.0)	0.00 (0.00–2.40)	0.244	0.625
Fatigue	Before	35 (38.5)	48 (53.3)	0.55 (0.29–1.03)	0.053	0.138	9 (9.9)	13 (14.4)	0.65 (0.23–1.76)	0.373	0.526
	After	23 (38.3)	27 (45.0)	0.76 (0.34–1.67)	0.579	0.752	5 (8.3)	10 (16.7)	0.46 (0.11–1.59)	0.269	0.625
Dizziness	Before	28 (30.8)	24 (26.7)	1.22 (0.61–2.46)	0.623	0.736	1 (1.1)	0 (0.0)	​	1.000	1.000
	After	20 (33.3)	14 (23.3)	1.64 (0.68–4.00)	0.311	0.550	1 (1.7)	0 (0.0)	​	1.000	1.000
Rash	Before	10 (11.0)	18 (20.0)	0.50 (0.19–1.22)	0.104	0.225	0 (0.0)	3 (3.3)	0.00 (0.00–2.38)	0.121	0.324
	After	6 (10.0)	15 (25.0)	0.34 (0.10–1.01)	0.053	0.244	0 (0.0)	2 (3.3)	0.00 (0.00–5.31)	0.496	0.625
Peripheral sensory neuropathy	Before	8 (8.8)	7 (7.8)	1.14 (0.34–3.88)	1.000	1.000	0 (0.0)	2 (2.2)	0.00 (0.00–5.26)	0.246	0.501
	After	3 (5.0)	6 (10.0)	0.48 (0.07–2.37)	0.491	0.709	0 (0.0)	2 (3.3)	0.00 (0.00–5.31)	0.496	0.625

AST, aspartate aminotransferase; ALT, alanine aminotransferase; OR, odds ratio; CI, confidence interval; FDR, false discovery rate; PSM, propensity score matching; THPyro, taxanes plus trastuzumab plus pyrotinib; THP, taxanes plus trastuzumab plus pertuzumab.

Data are presented as n (%). Percentages are calculated based on the number of patients in each group before (THP, n = 90; THPyro, n = 91) or after (n = 60 per group) PSM, as indicated in the respective table sections. Between-group comparisons for categorical variables (e.g., adverse events) were performed using Fisher’s exact test. ORs with 95% CIs were calculated. P values were adjusted for multiple comparisons using the two-stage linear step-up procedure of Benjamini, Krieger, and Yekutieli to control the FDR. A two-sided FDR-adjusted P value < 0.05 was considered statistically significant.

Hematologic toxicity did not show a consistent excess with THPyro. The difference in any-grade neutropenia observed before matching was attenuated after matching, and other adverse events did not remain significantly different after adjustment for multiple comparisons. Overall, THPyro was primarily characterized by increased gastrointestinal toxicity rather than a broad increase in hematologic or hepatic adverse events.

### 3.6 Exploratory prognostic model for patients with brain metastases

Because brain metastasis was the most prominent potential effect modifier, an exploratory PFS prognostic model was developed in this subgroup. Treatment regimen, hormone receptor status, number of metastatic sites, and radiotherapy were retained after LASSO selection and incorporated into a nomogram for estimating 12- and 18-month PFS probabilities (Figure 4A). This exploratory nomogram was internally validated using 1,000 bootstrap resamples only, without an independent external validation cohort. It should therefore not be used for clinical decision-making until externally validated in a larger independent cohort.

**FIGURE 4:**
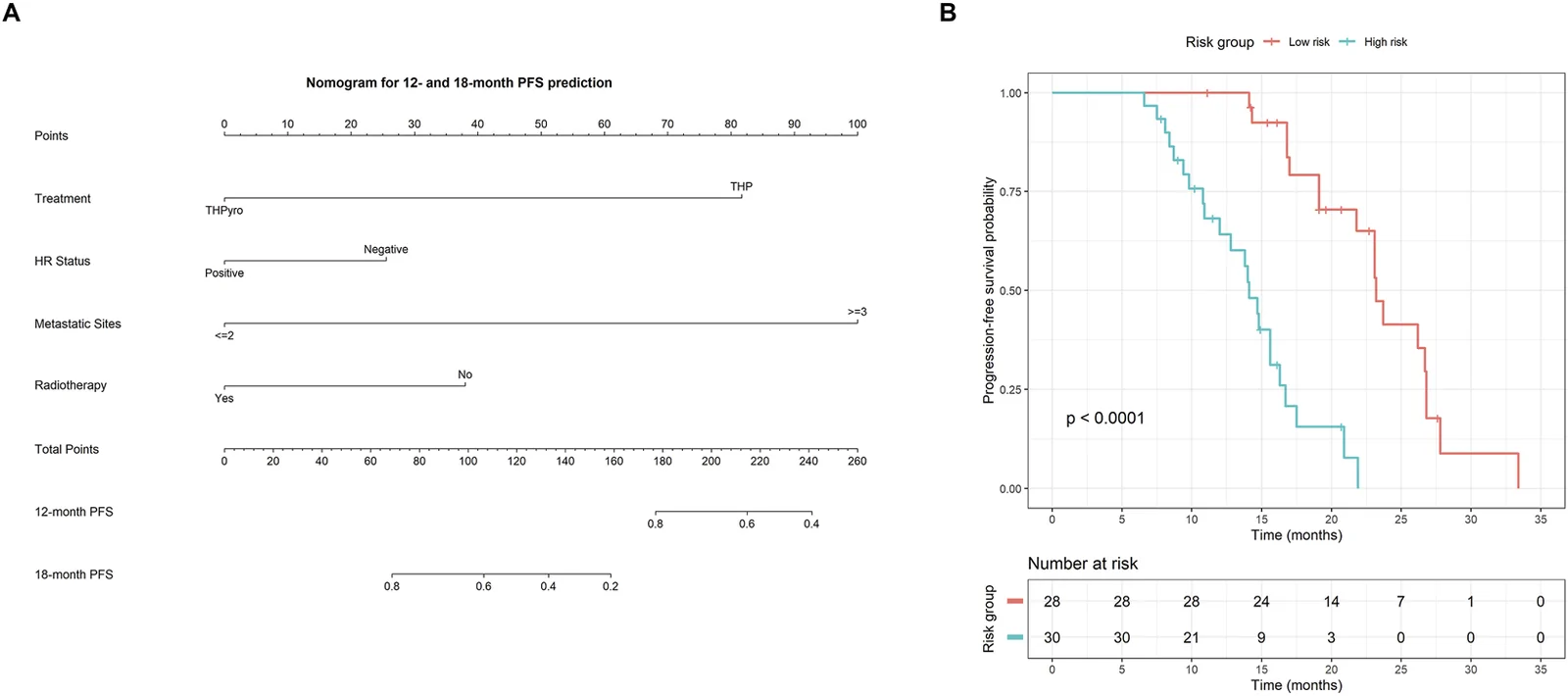
Exploratory prognostic model for PFS in patients with baseline brain metastases. **(A)** Nomogram for estimating 12- and 18-month PFS probabilities among patients with baseline brain metastases. The model incorporated treatment regimen, hormone-receptor status, number of metastatic sites, and receipt of radiotherapy. **(B)** Kaplan–Meier curves for PFS stratified by the median linear predictor of the prognostic model. This model was exploratory and internally validated only; therefore, it should not be used for clinical decision-making before external validation. PFS, progression-free survival; HR, hormone receptor; THPyro, taxane plus trastuzumab plus pyrotinib; THP, taxane plus trastuzumab plus pertuzumab.

In the multivariable Cox model, THP treatment and three or more metastatic sites were associated with poorer PFS, whereas radiotherapy was associated with a lower risk of progression (Table 4). The model showed good internal discrimination, with a Harrell C-index of 0.823 and an optimism-corrected C-index of 0.808. Risk stratification based on the median linear predictor separated patients into distinct PFS groups (Figure 4B). Additional internal validation results are presented in Supplementary Figure S7A–S7G.

**TABLE 4:** Multivariable Cox proportional hazards model for PFS in patients with brain metastases.

Variable	HR	95% CI	P value
Treatment regimen (THP vs THPyro)	6.036	2.551–14.279	<0.001
Hormone receptor status (Positive vs. Negative)	1.756	0.815–3.781	0.150
Number of metastatic sites (≥3 vs. <3)	9.032	3.189–25.579	<0.001
Radiotherapy (Yes vs. No)	0.433	0.200–0.938	0.034

HR, hazard ratio; CI, confidence interval; THPyro: taxanes plus trastuzumab plus pyrotinib; THP: taxanes plus trastuzumab plus pertuzumab; PFS: progression-free survival.

### 3.7 Exploratory prediction model for THPyro-associated diarrhea

Among 91 patients treated with THPyro, 26 (28.6%) developed grade≥2 diarrhea. An exploratory logistic regression model including baseline albumin level, ECOG performance status, and prophylactic loperamide use was developed and visualized as a nomogram (Figure 5A). This prediction model was likewise internally validated using 1,000 bootstrap resamples only and has not undergone external validation. Its clinical utility therefore remains unestablished pending validation in larger independent cohorts.

**FIGURE 5:**
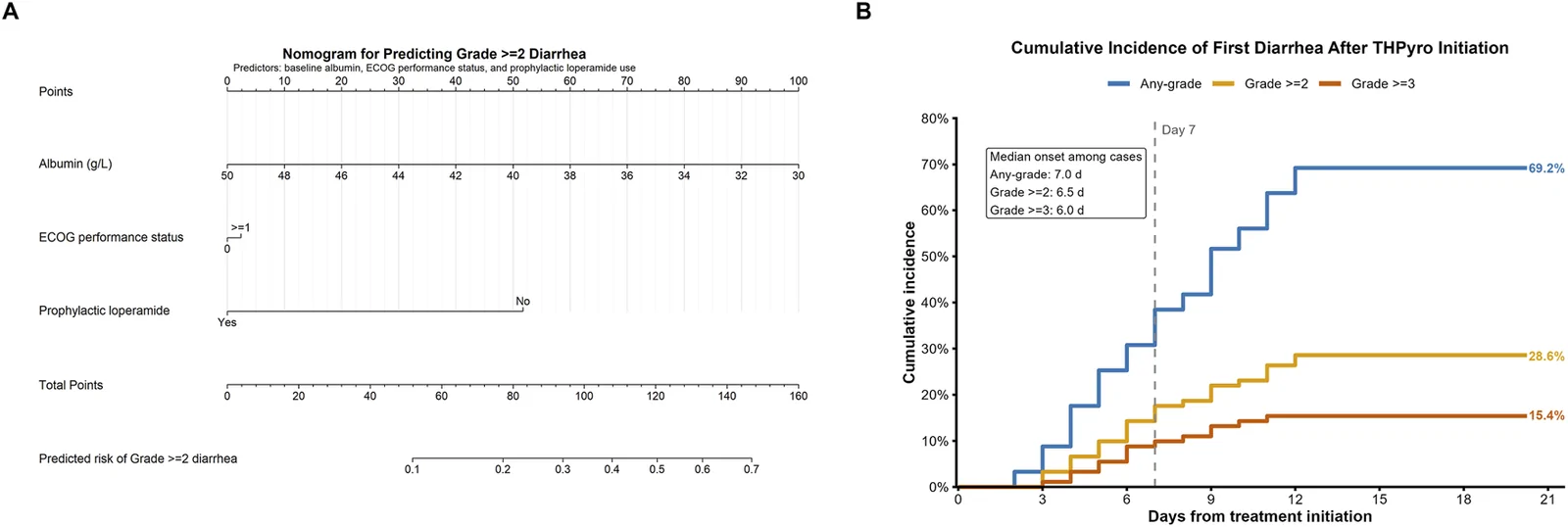
Exploratory prediction model for THPyro-associated grade ≥2 diarrhea. **(A)** Nomogram for predicting the risk of THPyro-associated grade ≥2 diarrhea based on baseline albumin level, ECOG performance status, and prophylactic loperamide use. **(B)** Cumulative incidence of first diarrhea event after THPyro initiation according to diarrhea severity. The dashed vertical line indicates day 7 after treatment initiation. Median onset times among patients with diarrhea were 7.0 days for any-grade diarrhea, 6.5 days for grade ≥2 diarrhea, and 6.0 days for grade ≥3 diarrhea. ECOG, Eastern Cooperative Oncology Group; THPyro, taxane plus trastuzumab plus pyrotinib.

Higher baseline albumin level (OR per 1 g/L increase, 0.852; 95% CI, 0.748–0.971; P = 0.016) and prophylactic loperamide use (OR, 0.191; 95% CI, 0.050–0.727; P = 0.015) were associated with a lower observed risk of grade≥2 diarrhea, whereas ECOG performance status was not significant (Table 5).

**TABLE 5:** Multivariable logistic regression model for THPyro-associated grade >=2 diarrhea.

Variable	OR	95% CI	P value
Baseline albumin (Per 1 g/L increase)	0.852	0.748–0.971	0.016
ECOG performance status (≥1 vs. 0)	1.078	0.356–3.265	0.894
Prophylactic loperamide use (yes vs. no)	0.191	0.050–0.727	0.015

OR, odds ratio; CI, confidence interval; ECOG, Eastern Cooperative Oncology Group; THPyro: taxanes plus trastuzumab plus pyrotinib.

The model showed moderate discrimination, with an apparent receiver operating characteristic (ROC) area under the curve (AUC) of 0.751 and an optimism-corrected AUC of 0.723 (Supplementary Figure S8A); calibration and decision curve analyses are shown in Supplementary Figure S8B, C. Diarrhea events occurred early after THPyro initiation, with median onset times of 7.0, 6.5, and 6.0 days for any-grade, grade≥2, and grade≥3 diarrhea, respectively (Figure 5B). Firth’s penalized logistic regression for grade≥3 diarrhea yielded directionally consistent but non-significant estimates (Supplementary Table S8).

## 4 Discussion

In this multicenter real-world cohort of patients with HER2-positive advanced breast cancer, first-line THPyro showed a PFS-related disease-control signal compared with THP after adjustment for baseline imbalances. PSM achieved well-balanced clinicopathologic characteristics between treatment groups, including age, menopausal status, performance status, hormone-receptor status, Ki-67, disease presentation, TNM stage, previous trastuzumab exposure, and metastatic sites. Within this balanced population, THPyro was associated with numerically longer PFS, an early separation of survival curves, and a significant gain in RMST, whereas ORR and DCR were comparable between groups. These findings suggest that the potential benefit of THPyro in routine practice may be reflected more by prolongation of PFS-related disease control than by a marked increase in initial radiographic response. This pattern is directionally consistent with prior Chinese real-world comparisons but also highlights meaningful between-cohort differences. In the 2025 multicenter study by Liu et al. (2025), THPy was associated with a numerically longer matched median PFS than THP (24.33 vs. 14.50 months; log-rank P = 0.309) together with a higher ORR after matching (78.9% vs. 57.9%; P = 0.048). In the larger 2026 multicenter cohort reported by Kuang et al. (2026), THPy was associated with a significant matched PFS benefit (23.6 vs. 17.3 months; HR, 0.68; P = 0.033), whereas the ORR difference was no longer significant after matching. Our findings therefore align more closely with the latter study in showing separation in time-to-event outcomes without a reproducible ORR advantage.

The discrepancy between similar response rates and improved time-to-event outcomes is clinically plausible. Response-based endpoints mainly capture early tumor shrinkage, while PFS integrates both initial antitumor activity and the durability of disease suppression. Notably, in the present study, neither the log-rank test nor the stratified Cox model demonstrated a statistically significant difference in PFS between the treatment groups, whereas the RMST analysis showed a significant 2.41-month gain in restricted mean PFS through 24 months. This discrepancy may be partly explained by the time-varying pattern of the treatment effect. The PFS curves showed greater between-group separation earlier during follow-up (12-month PFS, 96.7% vs. 81.6%) but converged by 24 months (25.0% vs. 25.4%). Moreover, the proportional-hazards assumption was mildly violated. Under such a pattern, a single hazard ratio may not fully capture the treatment effect across the entire follow-up period, while the overall log-rank comparison may also be influenced by the later convergence of the survival curves. In contrast, RMST quantifies the average progression-free time accumulated within a prespecified time horizon without requiring the proportional-hazards assumption. Thus, the early separation of the PFS curves could contribute to a significant difference in 24-month RMST. The RMST analysis therefore provided an absolute and clinically interpretable measure of treatment benefit over the prespecified 24-month horizon and served as a complementary analysis in the setting of potential non-proportional hazards. Recent statistical literature has emphasized the value of RMST-based estimands when hazards are non-proportional or when a clinically interpretable absolute survival time difference is needed (Alger et al., 2026; Wu et al., 2026). The wide CI around the Cox estimate and the lack of statistical significance in the Cox and log-rank analyses indicate that the observed RMST benefit should be interpreted as a signal of potential time-limited PFS benefit rather than definitive evidence of sustained treatment superiority. Compared with the PHILA trial, which demonstrated a clear randomized PFS and OS advantage for pyrotinib plus trastuzumab and docetaxel over placebo plus trastuzumab and docetaxel (Ma et al., 2026), our study addresses a different clinical question by comparing two active first-line anti-HER2 strategies in routine practice. The weaker statistical separation observed here is therefore not unexpected and underscores the importance of distinguishing randomized efficacy against placebo from comparative effectiveness between active regimens in heterogeneous real-world settings.

A notable finding was the treatment-effect signal among patients with baseline brain metastases. CNS involvement remains a major therapeutic challenge in HER2-positive metastatic breast cancer because of both the frequency of brain metastases and the limited intracranial penetration of many large-molecule agents. The HER2CLIMB trial established the clinical relevance of tucatinib in patients with brain metastases, demonstrating that the addition of tucatinib to trastuzumab and capecitabine significantly improved PFS and OS compared with placebo plus trastuzumab and capecitabine (Curigliano et al., 2022). A systematic review and meta-analysis further reported evidence supporting the activity of HER2-directed tyrosine kinase inhibitors in HER2-positive breast cancer brain metastases (Yu et al., 2023). Recent clinical reviews increasingly position systemic CNS-active therapy as an essential component of treatment sequencing for this population (Criscitiello et al., 2023; Rader et al., 2023). Notably, this subgroup pattern differs from prior head-to-head Chinese real-world studies, in which the most prominent exploratory signals involved prior neoadjuvant therapy (Liu et al., 2025) or *de novo* stage IV disease and liver metastasis (Kuang et al., 2026) rather than brain metastasis. This difference may reflect variation in case mix, sample size, or regional practice patterns across cohorts, but it also argues against overgeneralizing our subgroup result. Because CNS imaging was not protocol-mandated and local therapy was not standardized, this analysis cannot distinguish systemic extracranial disease control from true intracranial activity. Accordingly, the brain-metastasis subgroup result should be interpreted as exploratory rather than definitive evidence of intracranial efficacy.

The safety profile observed in this study also has practical implications. THPyro was associated with a higher incidence of diarrhea than THP, including grade 3 or higher diarrhea, while other hematologic and non-hematologic adverse events were generally manageable. This pattern is consistent with the known toxicity profile of irreversible HER-family tyrosine kinase inhibitors and with recent real-world evidence from Xinjiang evaluating first-line pyrotinib-based therapy (Xiao et al., 2025). The clinical importance of diarrhea extends beyond symptom burden because severe or persistent events may lead to dose interruption, dose reduction, dehydration, nutritional compromise, and reduced treatment adherence. In the present analysis, the exploratory diarrhea model identified clinically accessible variables, including loperamide use and albumin level, that may help stratify patients at higher risk. These findings support proactive toxicity management, early antidiarrheal intervention, hydration and nutritional monitoring, and individualized follow-up intensity when THPyro is selected.

The prognostic model for patients with brain metastases and the diarrhea risk model should be viewed as exploratory tools rather than ready-to-implement clinical decision instruments. Recent work has highlighted the potential value of nomogram-based approaches for estimating brain metastasis-related outcomes in breast cancer (Wang et al., 2025). Nevertheless, prediction models derived from small retrospective cohorts are vulnerable to optimism, variable-selection instability, and limited transportability. Both exploratory nomograms in the present study were internally validated using 1000-bootstrap resampling only, and no independent external validation cohort was available. Therefore, these nomograms should not be used for clinical decision-making or direct clinical application until their performance has been externally validated in larger, independent cohorts, preferably from the same geographic region and clinical setting.

This study has several strengths. It provides a head-to-head comparison of THPyro and THP in a regional real-world population, uses propensity score-based methods to reduce baseline imbalance, reports conventional response and survival endpoints together with RMST, and evaluates both subgroup-specific efficacy and treatment-associated toxicity. These features make the findings relevant to routine decision-making in settings where both pertuzumab-based dual antibody therapy and pyrotinib-based regimens may be available. The present cohort also adds complementary value to the existing literature because it is a geographically bounded six-center Xinjiang cohort rather than a broadly pooled national dataset, and because it extends the prior Xinjiang report of first-line pyrotinib efficacy and safety without a direct THP comparator (Xiao et al., 2025) by introducing a head-to-head regional comparison. Even if its overall direction is consistent with larger Chinese studies (Kuang et al., 2026; Liu et al., 2025), this region-specific dataset helps assess whether those signals remain observable in a distinct practice environment. At the same time, several limitations should be considered. The retrospective design cannot eliminate residual confounding, and treatment selection may have been influenced by physician preference, drug access, comorbidity, tumor burden, or patient socioeconomic factors. The matched cohort remained relatively small, which may have limited the precision and generalizability of the estimated treatment effect. Although the observed number of PFS events provided adequate nominal power to detect an effect of the observed magnitude, the confidence interval around the Cox estimate remained wide, and the proportional-hazards assumption showed a mild departure from proportionality. Therefore, the comparative PFS findings should be interpreted cautiously rather than as definitive evidence of treatment superiority. The OS data remained immature because of the limited number of observed death events, and the OS analysis was exploratory and underpowered. Accordingly, these data are insufficient to determine whether THPyro provides an OS benefit over THP. In addition, radiologic assessment intervals, intracranial disease evaluation, and adverse event reporting may have varied across centers. Because study center was not modeled explicitly, center-level confounding could not be fully addressed. The brain-metastasis subgroup analysis should also be interpreted with caution. The subgroup was relatively small, and treatment allocation was non-randomized, leaving the possibility of residual or unmeasured confounding despite adjustment for baseline imbalances. Moreover, the global Schoenfeld test for the adjusted interaction model indicated evidence of non-proportional hazards (P = 0.009), suggesting that the treatment effect may not have been constant over time. Therefore, although the sensitivity analyses yielded consistent treatment-effect estimates, the observed treatment-by-brain-metastasis interaction should be considered exploratory and hypothesis-generating rather than definitive evidence of better intracranial efficacy of THPyro. Prospective studies with protocol-mandated standardized CNS imaging assessments and coordination of local brain-directed therapy are warranted to validate this finding. Six patients were excluded because of treatment discontinuation for financial reasons. However, comprehensive socioeconomic and health-insurance information was not systematically collected in the current dataset; therefore, whether financial barriers influenced the choice between THPyro and THP could not be reliably assessed. This may have introduced unmeasured socioeconomic confounding and should be considered when interpreting the findings.

## 5 Conclusion

In conclusion, first-line THPyro was associated with a non-significant trend toward longer PFS and a significant 24-month RMST gain compared with THP in this retrospective matched cohort. An exploratory signal was observed among patients with baseline brain metastases, while diarrhea was more frequent and required proactive management. Prospective comparative studies with standardized CNS assessment, longer OS follow-up, and external validation of prediction models are needed.

## Data Availability

The original contributions presented in the study are included in the article/Supplementary Material, further inquiries can be directed to the corresponding author.
