# Supplementary material for: Real-world comparative outcomes of pyrotinib-based versus pertuzumab-based first-line therapy in HER2-Positive advanced breast cancer: a multicenter retrospective cohort study from Xinjiang, China
Source: Front Pharmacol. 2026 Oct 15;17:1943895. doi: 10.3389/fphar.2026.1943895 (PMC13716800; doi:10.3389/fphar.2026.1943895)
Supplement: Supplementary file 1 [file Supplementaryfile1.docx]

**Supplementary Table 1.** Landmark PFS rates before and after PSM.

| **Analysis** | **Time** | **THPyro** | **THP** | **ARR_pct** | **NNT_or_NNH** | **Logrank_P** |
| --- | --- | --- | --- | --- | --- | --- |
| Before PSM | 6 | 100.0% (100.0-100.0) | 98.9% (96.7-100.0) | -1.1% | NNH=91 | 0.187 |
| Before PSM | 12 | 95.6% (91.5-99.9) | 84.0% (76.3-92.4) | -11.6% | NNH=9 | 0.187 |
| Before PSM | 24 | 32.6% (23.2-45.9) | 33.1% (23.5-46.5) | 0.4% | NNT=231 | 0.187 |
| After Matching | 6 | 100.0% (100.0-100.0) | 98.3% (95.1-100.0) | -1.7% | NNH=60 | 0.087 |
| After Matching | 12 | 96.7% (92.2-100.0) | 81.6% (71.9-92.7) | -15.1% | NNH=7 | 0.087 |
| After Matching | 24 | 25.0% (15.5-40.5) | 25.4% (15.2-42.6) | 0.4% | NNT=251 | 0.087 |

THPyro: taxanes plus trastuzumab plus pyrotinib; THP: taxanes plus trastuzumab plus pertuzumab; PFS: progression-free survival; PSM: propensity score matching; ARR_pct: absolute risk reduction percentage; NNT: number needed to treat; NNH: number needed to harm.

ARR_pct and NNT_or_NNH are retained as exported from the original analysis file.

**Supplementary Table 2.** Conditional Cox model and time-varying treatment effect analysis after PSM.

| **Model** | **HR** | **CI_lower** | **CI_upper** | **P_value** | **PH_Global_P** | **TT_Coefficient** | **TT_P_value** |
| --- | --- | --- | --- | --- | --- | --- | --- |
| Conditional Cox (strata subclass) | 0.478 | 0.216 | 1.061 | 0.070 | 0.045 | 1.687 | 0.299 |

HR: hazard ratio; CI: confidence interval; PH: proportional hazards; TT: time-treatment interaction; PSM: propensity score matching.

**Supplementary Table 3.** OS summary and landmark OS rates before and after PSM.

| **Dataset** | **Overall N** | **Overall deaths, n (%)** | **Median follow-up, months** | **Maximum follow-up, months** | **Group** | **12-month OS, % (95% CI)** | **24-month OS, % (95% CI)** | **36-month OS, % (95% CI)** | **Log-rank P** |
| --- | --- | --- | --- | --- | --- | --- | --- | --- | --- |
| Before PSM | 181 | 20 (11.0) | 28.5 | 69.3 | THP | 100 (100–100) | 98.5 (95.7–100) | 92.7 (86.0–99.9) | 0.108 |
| Before PSM | 181 | 20 (11.0) | 28.5 | 69.3 | THPyro | 98.9 (96.8–100) | 92.9 (87.0–99.2) | 78.0 (65.7–92.5) |  |
| After PSM | 120 | 13 (10.8) | 28.4 | 69.3 | THP | 100 (100–100) | 97.8 (93.6–100) | 91.4 (82.5–100) | 0.376 |
| After PSM | 120 | 13 (10.8) | 28.4 | 69.3 | THPyro | 98.3 (95.1–100) | 94.3 (88.2–100) | 81.1 (67.2–98.0) |  |

OS, overall survival; CI: confidence interval; PSM, propensity score matching; THP: taxanes plus trastuzumab plus pertuzumab; THPyro, taxanes plus trastuzumab plus pyrotinib.

Log-rank P values indicate between-group comparisons within each dataset.

Values are presented as n (%) or survival probability (95% confidence interval).

**Supplementary Table 4.** Subgroup analysis of PFS in the IPTW-weighted cohort.

| **Subgroup** | **Level** | **HR** | **95% CI** | **P for interaction** |
| --- | --- | --- | --- | --- |
| Age (years) | <50 years | 0.833 | 0.429-1.618 | 0.569 |
| Age (years) | ≥50 years | 0.657 | 0.412-1.05 | 0.569 |
| Ki-67 | >20% | 0.593 | 0.383-0.919 | 0.157 |
| Ki-67 | ≤20% | 1.138 | 0.519-2.498 | 0.157 |
| Visceral metastasis | No | 1.014 | 0.405-2.539 | 0.354 |
| Visceral metastasis | Yes | 0.626 | 0.415-0.946 | 0.354 |
| Brain metastasis | No | 0.943 | 0.595-1.494 | <0.001 |
| Brain metastasis | Yes | 0.156 | 0.074-0.327 | <0.001 |
| Bone metastasis | No | 0.718 | 0.416-1.24 | 0.895 |
| Bone metastasis | Yes | 0.682 | 0.394-1.18 | 0.895 |
| ECOG performance status | 0–1 | 0.774 | 0.508-1.18 | 0.426 |
| ECOG performance status | ≥2 | 0.503 | 0.192-1.32 | 0.426 |
| Disease characteristic | De novo stage IV | 0.936 | 0.594-1.474 | 0.063 |
| Disease characteristic | Recurrent disease | 0.405 | 0.194-0.848 | 0.063 |
| Hormone receptor status | Negative | 0.756 | 0.442-1.294 | 0.743 |
| Hormone receptor status | Positive | 0.667 | 0.392-1.133 | 0.743 |
| Other metastasis | No | 0.947 | 0.609-1.473 | 0.138 |
| Other metastasis | Yes | 0.524 | 0.277-0.993 | 0.138 |
| Previous trastuzumab therapy | No | 0.846 | 0.541-1.322 | 0.210 |
| Previous trastuzumab therapy | Yes | 0.472 | 0.217-1.03 | 0.210 |
| Treatment | THPyro | 0.711 | 0.485-1.041 | 0.079 |

PFS: progression-free survival; IPTW: inverse probability of treatment weighting; THPyro, taxanes plus trastuzumab plus pyrotinib; HR: hazard ratio; ECOG: Eastern Cooperative Oncology Group.

**Supplementary Table 5.** Baseline characteristics and PFS event distribution among patients with baseline brain metastases.

| **Variable** |  | **Treatment Group** | | **P value** |
| --- | --- | --- | --- | --- |
|  | **N** | **THPyro**  **N=38** | **THP**  **N=20** |  |
| Age | 58 |  |  | 0.882 |
| <55 |  | 25 (66%) | 12 (60%) |  |
| ≥55 |  | 13 (34%) | 8 (40%) |  |
| ECOG score | 58 |  |  | >0.999 |
| 0–1 |  | 32 (84%) | 17 (85%) |  |
| ≥2 |  | 6 (16%) | 3 (15%) |  |
| Hormone receptor status | 58 |  |  | 0.808 |
| Positive |  | 16 (42%) | 7 (35%) |  |
| Negative |  | 22 (58%) | 13 (65%) |  |
| Ki-67 | 58 |  |  | 0.353 |
| ≤20% |  | 6 (16%) | 6 (30%) |  |
| >20% |  | 32 (84%) | 14 (70%) |  |
| Number of metastasis sites | 58 |  |  | 0.227 |
| ≤2 |  | 15 (39%) | 4 (20%) |  |
| ≥3 |  | 23 (61%) | 16 (80%) |  |
| Accept radiotherapy | 58 |  |  | 0.007 |
| No |  | 17 (45%) | 17 (85%) |  |
| Yes |  | 21 (55%) | 3 (15%) |  |
| Number of intracranial lesions | 58 |  |  | 0.726 |
| 1–2 |  | 26 (68%) | 12 (60%) |  |
| ≥3 |  | 12 (32%) | 8 (40%) |  |
| PFS event | 58 |  |  | 0.826 |
| Censor |  | 12 (32%) | 5 (25%) |  |
| Event |  | 26 (68%) | 15 (75%) |  |

THP: taxanes plus trastuzumab plus pertuzumab; THPyro, taxanes plus trastuzumab plus pyrotinib; PFS: progression-free survival; ECOG: Eastern Cooperative Oncology Group.

Data are presented as n (%).

P values were calculated using the chi-square test or Fisher’s exact test, as appropriate.

**Supplementary Table 6.** Sensitivity analysis of the treatment-by-brain-metastasis interaction for PFS after adjustment for baseline imbalances.

| **Analysis** | **Brain metastases, HR (95% CI)** | **No brain metastases, HR (95% CI)** | **P for interaction** |
| --- | --- | --- | --- |
| Original IPTW analysis | 0.156 (0.074-0.327) | 0.943 (0.595-1.494) | <0.001 |
| Adjusted IPTW analysis* | 0.163 (0.085-0.312) | 0.942 (0.581-1.526) | <0.001 |

PFS: progression-free survival; IPTW: inverse probability of treatment weighting; HR: hazard ratio; CI: confidence interval.

*: adjusted for brain-directed radiotherapy, number of intracranial lesions, and number of metastatic sites.

**Supplementary Table 7.** Additional sensitivity analyses of the association between treatment and PFS among patients with baseline brain metastases.

| **Analysis** | **HR (95% CI)** | **P value** |
| --- | --- | --- |
| Unadjusted Cox model | 0.214 (0.105-0.435) | <0.001 |
| Multivariable Cox model* | 0.092 (0.036-0.233) | <0.001 |
| IPTW-weighted Cox model* | 0.099 (0.036-0.273) | <0.001 |

PFS: progression-free survival; IPTW: inverse probability of treatment weighting; HR: hazard ratio; CI: confidence interval.

*: adjusted for brain-directed radiotherapy, number of intracranial lesions, and number of metastatic sites.

**Supplementary Table 8.** Sensitivity analysis using Firth’s penalized logistic regression for grade >=3 diarrhea.

| **Variable** | **OR (95% CI)** | **P value** |
| --- | --- | --- |
| Albumin (per 1 g/L increase) | 0.891 (0.766-1.024) | 0.105 |
| ECOG ≥1 vs 0 | 1.692 (0.475-6.173) | 0.415 |
| Prophylactic loperamide: Yes vs No | 0.519 (0.124-1.948) | 0.336 |

ECOG: Eastern Cooperative Oncology Group; OR: odds ratio; CI: confidence interval.
